# Neuromyelitis optica spectrum disorder overlapping with Sjögren’s disease: immunopathological links and therapeutic implications

**DOI:** 10.3389/fimmu.2026.1931729

**Published:** 2026-08-17

**Authors:** Wanfeng Xu, Haoyue Xing, Ling Hou

**Affiliations:** 1 Department of Endocrinology, Shengjing Hospital of China Medical University, Shenyang, China; 2 Department of Pediatrics, Shengjing Hospital of China Medical University, Shenyang, China

**Keywords:** aquaporin-4 antibody, autoimmune overlap phenotype, B-cell hyperactivity, blood-brain barrier dysfunction, interleukin-6 signaling, neuromyelitis optica spectrum disorder, Sjögren’s disease

## Abstract

Neuromyelitis optica spectrum disorder (NMOSD) is an autoimmune disease that mostly affects the central nervous system, and Sjögren’s disease (SjD) is an autoimmune disease that mostly affects the exocrine glands. Recent evidence indicates that these conditions frequently coexist and may have overlapping immunopathogenic mechanisms. Patients with NMOSD often exhibit serological or clinical features of SjD, and patients with SjD may develop inflammatory demyelinating events resembling NMOSD. This overlap can pose significant diagnostic and therapeutic challenges. Our narrative review synthesizes recent evidence on the epidemiology, clinical characteristics, immunopathogenic links, diagnostic considerations, and therapeutic implications of NMOSD-SjD overlap. This overlap phenotype is characterized by a diverse autoantibody profile, B-cell hyperactivity, cytokine dysregulation (particularly interleukin-6 and type I interferon signaling), and loss of immune tolerance. Blood-brain barrier dysfunction may enable circulating pathogenic antibodies and inflammatory mediators to access the central nervous system, although direct evidence for this in the overlap disease remains limited. We propose a structured diagnostic approach that integrates clinical phenotype, antibody testing, neuroimaging, and systemic autoimmune assessment to distinguish NMOSD-SjD overlap from SjD-related central nervous system involvement and myelin oligodendrocyte glycoprotein antibody-associated disease. We also describe therapeutic strategies that emphasize prevention of NMOSD relapse, selection of biologic treatments, assessment of systemic safety, and avoidance of misclassification. Recognition of NMOSD-SjD overlap has important implications for personalized diagnosis, assessment of systemic risk, and long-term management, and illustrates the relationship of systemic autoimmunity with organ-specific antibody-mediated central nervous system disease.

## 1 Introduction

Neuromyelitis optica spectrum disorder (NMOSD) is a severe autoimmune inflammatory disease of the central nervous system (CNS), in which patients experience recurring episodes of optic neuritis, longitudinally extensive transverse myelitis, and area postrema syndrome (1, 2). Historically, neuromyelitis optica was defined primarily by optic neuritis and acute myelitis. The term “spectrum disorder” was introduced to include patients with more diverse CNS presentations, such as area postrema, brainstem, diencephalic, and cerebral syndromes, and NMOSD was subsequently used in the 2015 international consensus criteria (3, 4). The identification of pathogenic aquaporin-4 immunoglobulin G (AQP4-IgG) in a substantial proportion of patients indicates that NMOSD is an antibody-mediated astrocytopathy that is distinct from multiple sclerosis (MS) (4–6). Sjögren’s disease (SjD), is a prototypical systemic autoimmune disease driven by B-cell hyperactivity in which patients experience lymphocytic infiltration of exocrine glands, autoantibody production, and a wide spectrum of extraglandular manifestations (7, 8).

Studies during the past two decades have suggested that NMOSD may coexist with systemic autoimmune diseases, particularly SjD and systemic lupus erythematosus (SLE), at a rate greater than expected by chance (9–13). Thus, patients with NMOSD may have antinuclear antibodies, anti-SSA/Ro antibodies, or clinical features suggestive of sicca; similarly, patients with SjD may develop inflammatory demyelination of the CNS that closely resembles NMOSD (14–17). This leads to a question whose answer has important diagnostic and therapeutic implications: Does the coexistence of NMOSD and SjD merely reflect shared autoimmune susceptibility and surveillance bias, or does it define a clinically meaningful overlap phenotype (18, 19)?

The overlap of NMOSD and SjD presents diagnostic and classification challenges because CNS inflammation in SjD may represent NMOSD, whereas systemic SjD may remain unrecognized in patients presenting with NMOSD (20, 21). Misclassification may delay NMOSD-specific therapy, lead to inappropriate use of disease-modifying treatments for MS, or result in under-recognition of systemic autoimmune activity (22, 23).

Previous reviews have generally addressed NMOSD and SjD separately or considered SjD as one of several autoimmune comorbidities of NMOSD. Consequently, there has been limited integration of studies that examined SjD-specific evaluations with the mechanistic uncertainty of AQP4-IgG-positive astrocytopathy, and these can have implications for treatment (14, 24–26). An integrated clinical synthesis is therefore needed to distinguish association from causality to improve diagnosis and therapeutic decisions (13).

In this narrative review, we critically synthesize current evidence on the epidemiology, clinical characteristics, immunopathogenic links, diagnostic challenges, and therapeutic implications of NMOSD-SjD overlap, with particular emphasis on the limitations of current evidence, differential diagnosis, and individualized management of patients (18, 19, 27, 28).

## 2 Methods

This narrative review identified relevant publications by searches of PubMed, Web of Science, and Scopus from database inception to February 2026. The search terms included combinations of “neuromyelitis optica spectrum disorder,” “NMOSD,” “aquaporin-4,” “AQP4-IgG,” “Sjögren’s disease,” “Sjögren’s syndrome,” “anti-SSA/Ro,” “systemic autoimmune disease,” “connective tissue disease,” “myelin oligodendrocyte glycoprotein,” “MOGAD,” “B cells,” “BAFF,” “interleukin-6,” “type I interferon,” “complement,” and “blood-brain barrier.” Historical terms, including “Sjögren’s syndrome” and “primary Sjögren’s syndrome,” were retained as search terms to identify studies published using the earlier nomenclature. Additional relevant studies were identified from the reference lists of selected articles and recent consensus or guideline documents.

Priority was given to peer-reviewed articles written in English, including systematic reviews, cohort studies, population-based studies, case series, mechanistic studies, diagnostic criteria, and treatment guidelines. Single case reports were considered when they provided specific information on temporal relationships, described an atypical presentation, or examined therapeutic decision-making in NMOSD-SjD overlap. Publications were excluded if they did not address NMOSD, SjD, CNS demyelination, systemic autoimmunity, or relevant immunopathogenic or therapeutic mechanisms. Data were extracted for qualitative analysis, with a focus on epidemiology, serology, neurological phenotype, SjD-related systemic features, proposed mechanisms, diagnostic challenges, and treatment implications. The findings were synthesized thematically, with emphasis on areas of convergence, uncertainty, and clinical applicability. When available, numerical data on study design, sample size, diagnostic criteria, ascertainment direction, overlap frequency, serological and glandular features, temporal sequences, neurological phenotypes, and clinical outcomes were extracted and summarized (**Table 1**). Study-specific definitions of SjD were retained, and studies that included SjD in the context of SLE or another connective tissue disease were distinguished from those restricted to SjD without another systemic autoimmune disease when this information was available.

**Table 1:** Major clinical studies of NMOSD-SjD overlap and their ascertainment directions.^a,b,c^.

Study; country/region	Study design and population	NMOSD and SjD definitions	Ascertainment direction and frequency	Serological and glandular findings	Temporal sequence and neurological phenotype	Outcomes	Main limitations
A. NMOSD-based studies assessing SjD or SjD-related serological autoimmunity (10, 13, 17, 31–33, 47).							
Wu et al., 2025; China (17)	Single-center retrospective cohort of 155 adults with AQP4-IgG-positive NMOSD recruited during an acute attack; 70 had SjD and 85 did not	NMOSD: 2007 or 2015 Wingerchuk criteria plus AQP4-IgG positivity. SjD: 2012 ACR or 2016 ACR/EULAR criteria	NMOSD → SjD: 70/155 (45.2%)	NMOSD–SjD *vs.* NMOSD without SjD: ANA, 100% *vs.* 56.8%; anti-SSA/Ro, 90.0% *vs.* 30.7%; anti-SSB/La, 27.1% *vs.* 5.3%. All patients were AQP4-IgG-positive. Glandular findings were not reported in detail	Optic neuritis, 25.7%; myelitis, 48.6%; area postrema syndrome, 18.6% in the overlap group. Combined optic neuritis and myelitis was less frequent in SjD patients	Median interval to relapse: 19 *vs.* 34 months. Median time to EDSS 4.0: 24 *vs.* 36 months. Baseline EDSS was also higher in the overlap group	Highly selected patients, single-center study, AQP4-IgG-positive acute-attack cohort; other autoimmune diseases were excluded. The 45.2% frequency should not be interpreted as population prevalence
Akaishi et al., 2023; Japan (10)	Nationwide cross-sectional analysis of the Diagnosis Procedure Combination administrative database; 28,285,908 individuals from 1,203 hospitals, including 8,477 with NMOSD and 174,108 with SjD	NMOSD and SjD based on administrative diagnostic records; clinical criteria, antibody status, and specialist adjudication were unavailable	NMOSD → SjD: 986/8,477 (11.6%). SjD → NMOSD: 986/174,108 (0.57%). Odds Ratio for NMOSD in SjD: 21 (95% CI 20–23)	NR	NR	Relapse, disability and treatment outcomes were not assessed	Cross-sectional, code-based study without clinical adjudication, AQP4-IgG data, formal SjD classification, temporal information, or neurological outcome data
Lin et al., 2022; China (33)	Single-center retrospective cohort of 102 NMOSD patients; 37 were anti-SSA/Ro-positive and 65 were anti-SSA/Ro-negative	NMOSD: 2015 IPND criteria. The primary grouping was based on anti-SSA/Ro status rather than SjD	Anti-SSA/Ro positivity: 37/102 (36.3%). The frequency of SjD cannot be inferred from this antibody-stratified analysis	Positivity for AQP4-IgG, anti-SSB/La, ANA, and anti-dsDNA was more frequent in anti-SSA/Ro-positive patients.	At initial presentation, the prevalence of optic neuritis was 43/102 (42.1%) and the prevalence of transverse myelitis was 29/102 (28.4%). There was no clear disease-specific imaging phenotype according to anti-SSA/Ro status	Anti-SSA/Ro-positive *vs.* negative patients: median time to EDSS 4.0: 24 *vs.* 84 months; median time to relapse: 24 *vs.* 60 months	Retrospective single-center study; outcomes were analyzed according to anti-SSA/Ro positivity, not SjD; possible confounding by SLE and other systemic autoimmune diseases
B. SjD-based studies assessing NMOSD or AQP4-IgG							
Qiao et al., 2015; China (32)	Single-center retrospective cohort of 616 SjD patients treated between 1985 and 2013; 43 had NMO/NMOSD	SjD: revised American-European Consensus Group criteria. NMO/NMOSD: contemporaneous Wingerchuk criteria, including limited forms	SjD → NMO/NMOSD: 43/616 (7.0%); 21/43 had classical phenotype and 22/43 had limited phenotype	AQP4-IgG, 89.3%; anti-Ro/SSA or anti-La/SSB, 41/43 (95.3%). Dry mouth, 26/43 (60.5%); objective xerostomia, 31/43 (72.1%); dry eyes, 23/43 (53.5%); abnormal objective ocular test, 32/43 (74.4%). Positive labial salivary-gland biopsy, 28/33 (84.8%)	Neurological manifestations were an early or presenting feature in approximately 72%. Optic-nerve involvement, 32/43 (74.4%); spinal-cord involvement, 36/43 (83.7%); LETM, 32/43 (74.4%)	Long-term relapse, disability and comparative treatment outcomes: NR	Historical single-center referral cohort; older NMOSD definitions and antibody assays; possible inpatient, severity and referral bias
Afzali et al., 2023; Germany (47)	Exploratory cross-sectional tertiary-center study of 194 SjD patients; 19 had a CNS demyelinating phenotype	SjD: 2016 ACR/EULAR criteria. NMOSD: 2015 IPND criteria	AQP4-IgG-positive NMOSD: 2/194 overall and 2/13 among tested CNS patients. Three additional patients fulfilled criteria for AQP4-IgG-negative NMOSD	Positive anti-SSA/Ro, 15/19 (78.9%) of the CNS group. Only 2/19 (10.5%) reported sicca at first presentation; 8/17 (47.0%) developed sicca during follow-up. Positive salivary-gland biopsy, 14/16 (87.5%)	Neurological symptoms were as first SjD manifestation, 16/19 (84.2%). Myelitis as the index event, 13/19 (68.4%); optic neuritis as the index event, 5/19 (26.3%); both events during follow-up, 4/19 (21.1%)	Residual neurological deficits remaining, 18/19. Median EDSS, 2.0. Rituximab was associated with stable disease or improvement, 8/9	Small, highly selected tertiary-center CNS subgroup; AQP4-IgG was not tested in all patients; heterogeneous CNS phenotypes included MS-like disease, isolated demyelination and NMOSD
C. Systematic reviews and pooled evidence							
Esposito et al., 2024; international (13)	Systematic review and proportional random-effects meta-analysis of studies examining NMOSD and systemic connective-tissue diseases	Study-specific NMOSD and SjD definitions; criteria and screening intensity varied among studies	Pooled prevalences: NMOSD among unselected SjD cohorts, 6.5% (95% CI 4.7–8.6%); NMOSD among neurologically selected SjD cohorts, 26.5% (95% CI 5.5–54.6%); SjD among NMOSD cohorts, ~ 7.0%	Detailed pooled estimates for AQP4-IgG, anti-SSA/Ro, anti-SSB/La and sicca were not available	Temporal sequence and individual neurological syndromes were not uniformly pooled	Relapse, disability and treatment outcomes were too heterogeneous for a unified estimate	Marked clinical and methodological heterogeneity; variable classification criteria; selected neurological cohorts generated substantially higher estimates than unselected SjD cohorts
Prasad et al., 2024; international (31)	Systematic review of individual-patient data from case reports and case series; 44 AQP4-IgG- or NMO-IgG-positive overlap cases	Inclusion required AQP4-IgG or NMO-IgG positivity and at least one manifestation of NMOSD and primary SjD (historical definition); formal classification was not uniform among reports	Frequency could not be estimated because only established overlap cases were included	All cases were AQP4-IgG- or NMO-IgG-positive. Total population for anti-SSA/Ro, anti-SSB/La, ANA and sicca were not uniformly available	NMOSD preceded SjD in 20/44 (45.5%), followed SjD in 13/44 (29.5%), and occurred simultaneously in 11/44 (25.0%). Transverse myelitis occurred in 31/44 (70.5%), optic neuritis in 21/44 (47.7%), cerebral syndrome in 14/44 (31.8%), brainstem syndrome in 10/44 (22.7%), and area postrema syndrome in 5/44 (11.4%)	Relapsing disease was reported in 27/44 (61.4%). At a median follow-up of 2.4 years, 39/44 (88.6%) improved, 3/44 (6.8%) stabilized and 2/44 (4.5%) worsened	Case-reports and publication bias; incomplete variables; heterogeneous historical definitions of SjD, treatments, and follow-up; no total population for prevalence estimation

ACR, American College of Rheumatology; ANA, antinuclear antibody; AQP4-IgG, aquaporin-4 immunoglobulin G; CI, confidence interval; CNS, central nervous system; EDSS, Expanded Disability Status Scale; EULAR, European Alliance of Associations for Rheumatology; IPND, International Panel for NMO Diagnosis; LETM, longitudinally extensive transverse myelitis; NMO, neuromyelitis optica; NMOSD, neuromyelitis optica spectrum disorder; NR, not reported; OR, odds ratio; SjD, Sjögren’s disease.

SjD terminology and diagnostic labels are reported as in the original studies. In the narrative text, the term “Sjögren’s disease” and the abbreviation “SjD” are used consistently; historical terms are retained only when describing study-specific definitions.

Anti-SSA/Ro positivity was recorded separately from SjD. Overlap frequencies were based on the study-specific definition or classification of SjD rather than seropositivity alone.

^a^ Historical terms, such as primary Sjögren’s syndrome and Sjögren’s syndrome, are reported as in the original studies.

^b^ Anti-SSA/Ro positivity alone was not considered equivalent to SjD. Studies primarily stratified according to anti-SSA/Ro status are presented separately from those requiring formal SjD classification.

^c^ Frequencies should be interpreted according to the direction of ascertainment, total population (denominator), and study setting. Estimates from neurologically selected, tertiary-center or acute-attack cohorts should not be interpreted as population prevalence.

## 3 Epidemiology and clinical overlap of NMOSD and Sjögren’s disease

### 3.1 Autoimmune comorbidities in NMOSD

Autoimmune comorbidities can occur in patients with NMOSD and other autoimmune diseases. For example, large cohort studies and systematic reviews consistently demonstrated that patients with NMOSD had greater prevalences of concomitant systemic autoimmune diseases than individuals in the general population and patients with MS (9–12, 29). SjD is one of the most common of these comorbidities (18, 19). Similarly, real-world data from multicenter cohorts demonstrated a greater prevalence of SjD in certain geographically distinct populations of patients with NMOSD (10). Importantly, this association was stronger in patients with AQP4-IgG-seropositive NMOSD, suggesting a possible immunological relationship (30, 31).

Population-based and national database studies have provided additional evidence regarding the prevalence of this overlap (13, 29). In particular, analyses of East Asian cohorts (which have higher prevalences of NMOSD and SjD) indicated that SjD may be the most common systemic autoimmune disease that occurs with NMOSD (30). This suggests that screening for systemic autoimmunity, particularly SjD, should be considered an integral part of evaluating NMOSD patients (18, 19). The reported frequencies of SjD among NMOSD cohorts vary substantially according to study design and patient selection. A meta-analysis estimated that approximately 7% of patients in NMOSD cohorts had SjD, whereas a Japanese administrative database reported SjD in 986 (11.6%) of 8,477 patients with NMOSD (10, 13). A substantially higher frequency was reported in a tertiary-center cohort study that examined AQP4-IgG-positive patients during acute attacks (17). These estimates should therefore be interpreted according to the total population (denominator), setting, and intensity of rheumatological assessment (**Table 1**).

### 3.2 Frequency of co-occurrence and temporal relationship of SjD and NMOSD

The clinical recognition of the NMOSD-SjD overlap phenotype can be complicated because previous studies found that SjD may occur before, the same time as, or after the diagnosis of NMOSD (31). It is important to consider the direction of ascertainment when interpreting overlap frequencies. Studies beginning with NMOSD cohorts estimate the proportion of patients who also have the criteria for SjD, whereas studies beginning with SjD cohorts estimate the proportion of patients who also have NMO/NMOSD or AQP4-IgG positivity. For example, a Chinese study of a cohort of SjD patients identified NMO/NMOSD in 43 of 616 patients (7.0%), and a meta-analysis estimated NMOSD in 6.5% of unselected SjD cohorts (13, 32). These estimates should not be directly compared with the frequency of SjD reported in cohorts of NMOSD patients. In an individual-patient-data systematic review of 44 patients with AQP4-IgG- or NMO-IgG-positive overlap, NMOSD preceded the diagnosis of SjD in 20 patients (45.5%), followed the diagnosis of SjD in 13 patients (29.5%), and was concurrent with the diagnosis of SjD in 11 patients (25.0%) (31). In many patients, systemic autoimmune features or anti-SSA/Ro antibodies (characteristic of SjD) are detected years before the first demyelinating event (characteristic of NMOSD), suggesting that systemic autoimmunity may precede disorders of the CNS (31, 33). In other patients, NMOSD may be the initial clinical diagnosis, and this is followed by the development of symptoms of sicca or the formal diagnosis of SjD after the onset of neurological disorders (18, 19). The recognition of SjD may be delayed because the symptoms of sicca may be mild, overlooked, or attributed to an adverse effect of medication, particularly in young patients (8, 34). Some patients with NMOSD and anti-SSA/Ro positivity who did not initially fulfill the classification criteria for SjD may subsequently develop additional glandular or systemic features (33, 35). However, anti-SSA/Ro positivity alone should be reported as serological autoimmunity or possible evolving SjD, not as equivalent to established NMOSD-SjD overlap.

Notably, patients with the NMOSD-SjD overlap do not always exhibit the classical glandular manifestations of SjD. Previous studies have identified patients with SjD who presented primarily with symptoms of the CNS but without overt sicca, emphasizing that the absence of sicca should not exclude an underlying systemic autoimmune process (15, 36, 37). The presence of such patients has important implications for diagnosing the overlap phenotype, and argues against reliance on symptoms alone when evaluating NMOSD patients for SjD (18, 19).

### 3.3 Clinical phenotype of patients with NMOSD-SjD overlap

Patients with NMOSD-SjD overlap appear to have a clinically recognizable phenotype that is not disease-specific. Because AQP4-IgG-positive NMOSD and SjD are more common in females, patient sex provides no useful information for recognition of the overlap phenotype. The more informative features are anti-SSA/Ro positivity, the timing of sicca or systemic autoimmune features relative to neurological onset, extraglandular manifestations, and pattern of relapse (10, 31). In one selected AQP4-IgG-positive cohort of patients with or without SjD, patients with SjD had a shorter median time to relapse (19 *vs.* 34 months) and a shorter median time to an Expanded Disability Status Scale (EDSS) score of 4.0 (24 *vs.* 36 months) (17). These findings require validation in independent and more diverse populations.

Some neurological studies showed that optic neuritis and longitudinally extensive transverse myelitis were the most common features in patients with the overlap phenotype, similar to classical NMOSD (38, 39). However, other neurological studies reported greater involvement of the area postrema and a greater burden of multifocal lesions in the overlap phenotype (30, 40). In addition, systemic manifestations, such as arthralgia, fatigue, Raynaud’s phenomenon, and hematologic abnormalities, may also occur, thus complicating diagnosis and treatment (18, 19).

**Figure 1:**
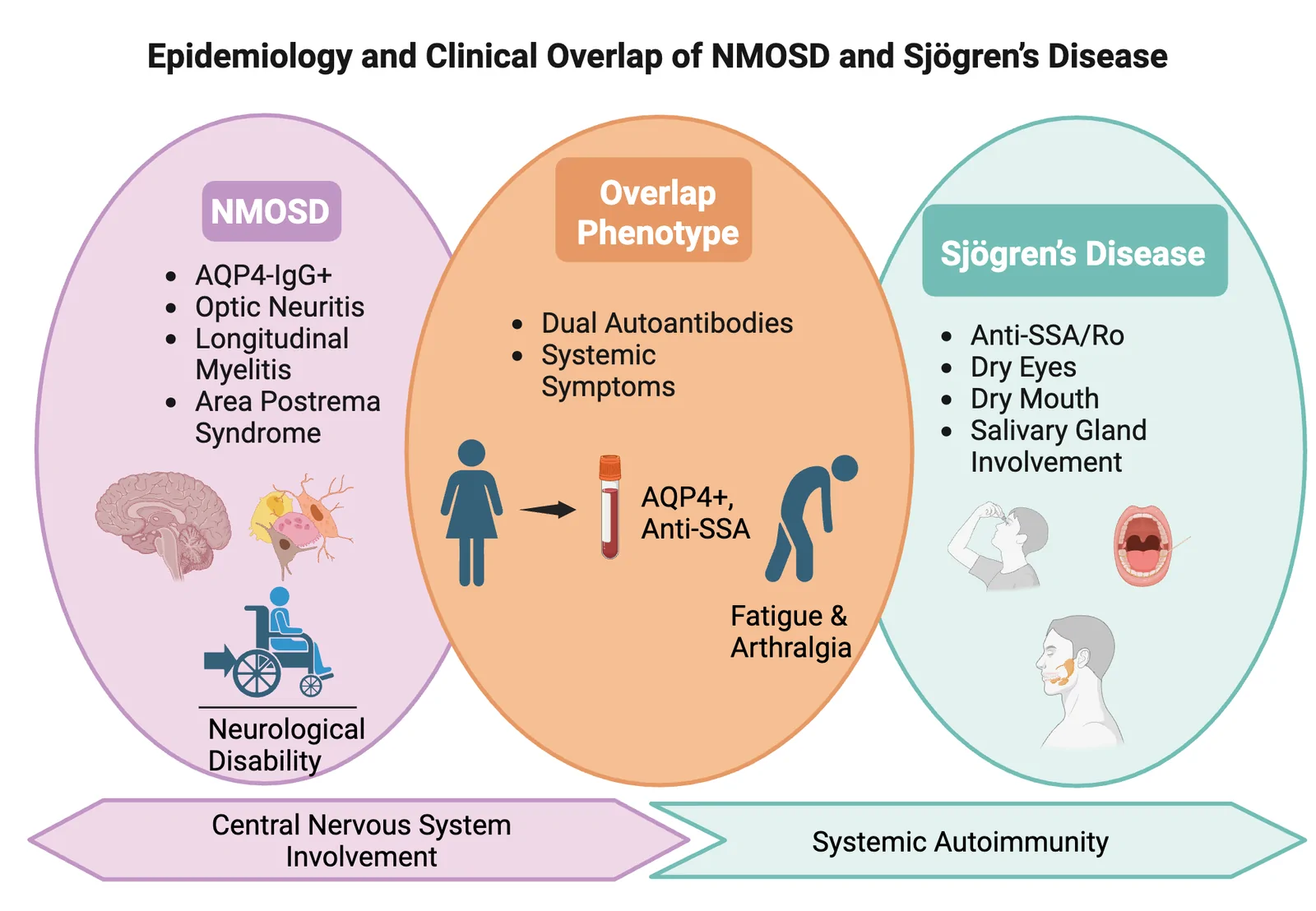
Epidemiological and clinical overlap of neuromyelitis optica spectrum disorder (NMOSD) and Sjögren’s disease (SjD). NMOSD patients have a high prevalence of systemic autoimmune comorbidities, especially SjD. Overlap patients are predominantly female and may present with SjD before, the same time as, or after the onset of NMOSD. The clinical features may include the typical manifestations of NMOSD (optic neuritis and longitudinally extensive transverse myelitis) and SjD (systemic autoimmunity). NMOSD-SjD overlap may be a clinically meaningful phenotype, although a causal relationship and disease-specific biomarkers remain to be established. Created in BioRender. Hou, L. (2026). https://BioRender.com/idlqiig.

Collectively, epidemiological and clinical data suggest that the co-occurrence of NMOSD and SjD in some patients is probably not attributable to chance, although a causal relationship remains unproven (40–43). Rather than defining a separate disease entity, current data support recognition of NMOSD-SjD overlap as a clinically meaningful phenotype that has important diagnostic and therapeutic implications (**Figure 1**). The principal quantitative studies, including their ascertainment direction, clinical findings, outcomes, and methodological limitations, are summarized in **Table 1**.

### 3.4 Strengths and limitations of current evidence

Multiple lines of evidence support an association between NMOSD and SjD, but the strength of this evidence is limited. Most overlap-specific data are from retrospective cohorts, studies of administrative databases, systematic reviews of heterogeneous reports, and case series, rather than prospective cohorts designed to examine NMOSD-SjD overlap as a predefined outcome. Because estimates of the prevalence, temporal sequence of different symptoms, risk of relapse, and burden of systemic disease vary among studies, the evidence supporting a causal relationship must be considered incomplete.

Several additional limitations should be considered. First, the sample sizes of studies that examined patients with confirmed NMOSD-SjD overlap were often small, particularly when analyses were restricted to AQP4-IgG-positive disease and formally classified as SjD. Second, many of the study cohorts were East Asian populations, and the epidemiology of NMOSD, AQP4-IgG seropositivity, and systemic autoimmune diseases may differ in Western populations. Third, there is a high likelihood of surveillance bias because patients with NMOSD, especially those with AQP4-IgG positivity, may undergo more extensive serological testing for autoimmune diseases, and patients with SjD who develop neurological symptoms may be preferentially evaluated for AQP4-IgG or MOG-IgG. This can increase the recognition of coexisting diseases, but does not prove a direct pathogenic link.

A related classification issue is the coexistence of SLE and other connective tissue diseases. A recent cohort study of 71 patients with NMOSD reported that 26 had a coexisting systemic autoimmune disease; SLE was the most common of these diseases and SjD was also present (21). A meta-analysis estimated NMOSD occurred in 0.6% of unselected SLE cohorts, but in 6.5% of unselected SjD cohorts (13). Because anti-SSA/Ro positivity, cytopenias, hypocomplementemia, and some extraglandular manifestations may occur in SLE and SjD, these findings should not be attributed to SjD without disease-specific clinical assessments. Studies and clinical evaluations should therefore distinguish SjD without another connective tissue disease from SjD with SLE or another autoimmune disease (historically termed “secondary SjD”), and should separately apply the relevant classification criteria for NMOSD, SjD, and SLE.

Therefore, the co-occurrence of NMOSD and SjD should be viewed as a clinically meaningful overlap phenotype rather than a proven independent disease. Shared autoimmune susceptibility, B-cell-dominant immune dysregulation, activation of cytokines, and complement-mediated astrocyte injury provide biologically plausible links, but overlap-specific biomarkers and prospective validation are still lacking. Recognition of these limitations is important, because it means that the association of these diseases can be used to increase diagnostic vigilance and provide therapeutic risk stratification, without overstating causality.

## 4 Immunopathogenic links between NMOSD and SjD: convergence and uncertainty

Before considering potential immunopathogenic links, it is important to distinguish the different types of evidence. AQP4-IgG-positive NMOSD is primarily an antibody-mediated astrocytopathy, whereas SjD is a heterogeneous systemic autoimmune disease in which CNS demyelination only occurs in a subset of patients. Unless otherwise specified, we derived the mechanistic evidence discussed below mainly from separate studies of NMOSD and SjD, rather than from patients with confirmed NMOSD-SjD overlap. The pathways described should therefore be interpreted as biologically plausible points of intersection, rather than evidence of a shared or unified pathogenesis.

### 4.1 B-cell hyperactivity

B-cell hyperactivity is an important immunopathogenic feature of SjD and is also related to the production of AQP4-IgG in NMOSD, although the cells and tissues associated with this response may differ between the two conditions (1, 34, 44). For patients with NMOSD, there is evidence that AQP4-IgG, which is produced by peripheral plasmablasts and plasma cells, leads to complement-mediated astrocyte injury and has a pivotal role in the pathogenesis of this disease (1, 5, 6, 44, 45). Similarly, patients with SjD have profound dysregulation of B cells, including hypergammaglobulinemia, production of autoantibodies, and an excess of autoreactive B-cell clones (7, 34).

A key commonality of these two diseases is elevated levels of B-cell activating factor (BAFF) (46, 47). In particular, BAFF overexpression occurs in SjD and correlates with the EULAR Sjögren’s syndrome disease activity index (ESSDAI), autoantibody titer, and ectopic germinal centers in the salivary glands (48, 49). BAFF overexpression in NMOSD can lead to prolonged survival and differentiation of AQP4-IgG-producing plasmablasts (48, 50). These findings suggest that BAFF-induced survival, maturation, and selection of B cells may contribute to sustained autoantibody production in these different autoimmune diseases, although direct evidence of these alterations in the overlap disease remains limited (46–48, 50).

Clinical observations support the importance of B-cell hyperactivity in patients with the overlap phenotype. These patients frequently present with production of a broader variety of autoantibodies, especially antinuclear antibodies and anti-SSA/Ro antibodies, in addition to AQP4-IgG (14, 31). This broad humoral immune response is consistent with a systemic B-cell-dominant autoimmune state. Consequently, NMOSD-SjD overlap may be considered a clinically meaningful condition in which systemic B-cell-dominant autoimmunity coexists with AQP4-IgG-mediated CNS injury, rather than proof of disease continuum mediated by B cells (18, 19).

### 4.2 IL-6 and type I interferon signaling

Patients with each disease also have dysregulated cytokine networks, although the evidence is not as strong for patients with the overlap phenotype (41, 42). Interleukin-6 (IL-6) is particularly relevant because it promotes plasmablast survival, increases permeability of the blood-brain barrier (BBB), and amplifies inflammatory responses in NMOSD (51–53). Other research showed that elevated levels of IL-6 in serum and cerebrospinal fluid (CSF) were associated with greater disease activity and risk of relapse in patients with NMOSD (12, 51).

IL-6 also contributes to the autoimmune response in SjD because it activates B cells, production of autoantibodies, and chronic glandular or systemic inflammation (34). The efficacy of IL-6 receptor blockade in NMOSD supports the pathogenic relevance of this pathway. However, there is currently no evidence that SjD-related systemic inflammatory activity predicts a benefit from IL-6 receptor blockade or should be used to guide treatment selection in patients with NMOSD-SjD overlap (51, 54).

Both diseases are also characterized by increased type I interferon (IFN) signaling. In fact, SjD is considered to have an ‘interferon signature’ because persistent activation of IFN-induced genes leads to immune dysregulation and tissue damage (52). There is also evidence that type I IFN pathways are activated in patients with NMOSD who have systemic autoimmunity or features of connective tissue disease (53). Although activation of type I IFN may contribute to impaired immune tolerance and amplification of systemic autoimmunity, its effect on the transition from SjD-related autoimmunity to AQP4-IgG-mediated CNS injury remains unproven (18, 19, 53).

### 4.3 Ectopic germinal centers and humoral immune tolerance

A characteristic pathological feature of SjD is the formation of ectopic germinal center-like structures (eGCs) within inflamed salivary glands. These eGCs function in B-cell maturation, somatic hypermutation, and production of autoantibodies (1, 55, 56), and are associated with higher disease activity, increased diversity of autoantibodies, and an increased risk of lymphoproliferative complications (49, 55). The development of eGCs is therefore an indication of a significant dysfunction of peripheral immune tolerance.

Whether eGC-like structures contribute to the generation or amplification of pathogenic antibodies in NMOSD remains uncertain. Although AQP4-IgG is apparently produced by peripheral plasmablasts and plasma cells, the precise sites of autoantibody generation and maturation are uncertain (12, 44, 50, 57). The co-occurrence of SjD with NMOSD in some patients raises the possibility that peripheral lymphoid niches may amplify humoral autoimmunity in some overlap patients, but there is no direct evidence that SjD-associated eGCs generate AQP4-IgG. In agreement, several studies have reported higher autoantibody titers and broader autoantibody profiles in NMOSD patients who had a coexisting systemic autoimmune disease (10, 11).

These observations support a cautious model in which failure of B-cell tolerance, increased BAFF-driven signaling, and peripheral plasmablasts and plasma-cell responses may provide a permissive background for sustained humoral autoimmunity in selected patients, but do not imply that SjD-associated salivary-gland eGCs generate AQP4-IgG (12, 34, 48–50, 57).

### 4.4 Complement, T cells, and innate immune amplification

In addition to B-cell pathways, it is also important to consider complement activation, T cells, and innate immune responses to understand the mechanism of NMOSD-SjD overlap. In AQP4-IgG-positive NMOSD, complement activation leads to astrocyte injury after AQP4-IgG binds to the astrocyte endfeet, and this leads to complement-dependent cytotoxicity, recruitment of inflammatory cells, and secondary injury of oligodendrocytes and neurons (6, 12, 44, 45, 58). This mechanism is central to the pathogenesis of NMOSD and provides a biological rationale for complement inhibition for prevention of relapse.

By contrast, complement abnormalities in SjD are more often indicators of increased systemic immune complex activity, extraglandular disease, cryoglobulinemia, or lymphoproliferation rather than direct mediators of astrocyte injury (34, 49). Thus, the complement system is dysregulated in both diseases and can be used for clinical risk stratification, but the role of SjD-related complement activation in triggering NMOSD attacks remains unproven.

T cells and innate immune cells may further amplify the dysregulation of the complement system. In SjD, epithelial cells, dendritic cells, monocytes, and T-cell subsets increase the production of type I IFN, antigen presentation, and B-cell signaling, leading to maintenance of chronic tissue inflammation (34, 52). In NMOSD, innate immune activation and T-cell-derived cytokines can increase the permeability of the BBB and facilitate the recruitment of granulocytes, macrophages, and complement-fixing antibodies into CNS lesions (12, 56, 57). This suggests that T-cell and innate immune pathways are best viewed as amplifiers of humoral autoimmunity and tissue injury, not that NMOSD and SjD share a single unified pathogenic pathway.

### 4.5 Blood-brain barrier dysfunction: a hypothetical ‘two-hit’ model

Despite the presence of systemic immune dysregulation, a key question remains: Why do only some patients with systemic autoimmunity develop AQP4-IgG-mediated CNS injury? Dysfunction of the BBB may be a permissive factor, because this can allow circulating AQP4-IgG and inflammatory mediators to access the CNS (12, 58–61). In particular, there is evidence that patients with NMOSD experience disruption of the BBB before astrocyte damage, and this allows circulating AQP4-IgG to bind aquaporin-4 in the CNS (58–61).

Systemic inflammation in SjD could theoretically increase endothelial activation and barrier vulnerability through cytokine-mediated mechanisms that include IL-6 and type I IFN signaling (34, 53, 57, 60–62). However, there is no direct evidence that SjD-related systemic inflammation disrupts the BBB or precipitates NMOSD attacks in patients with confirmed overlap. Therefore, the role of systemic inflammation as a ‘second hit’ should be regarded as a hypothesis, not an established mechanism. Although this hypothesis may provide a framework for future investigations, it requires prospective validation in overlap-specific studies.

### 4.6 Integrated mechanistic model

**Figure 2:**
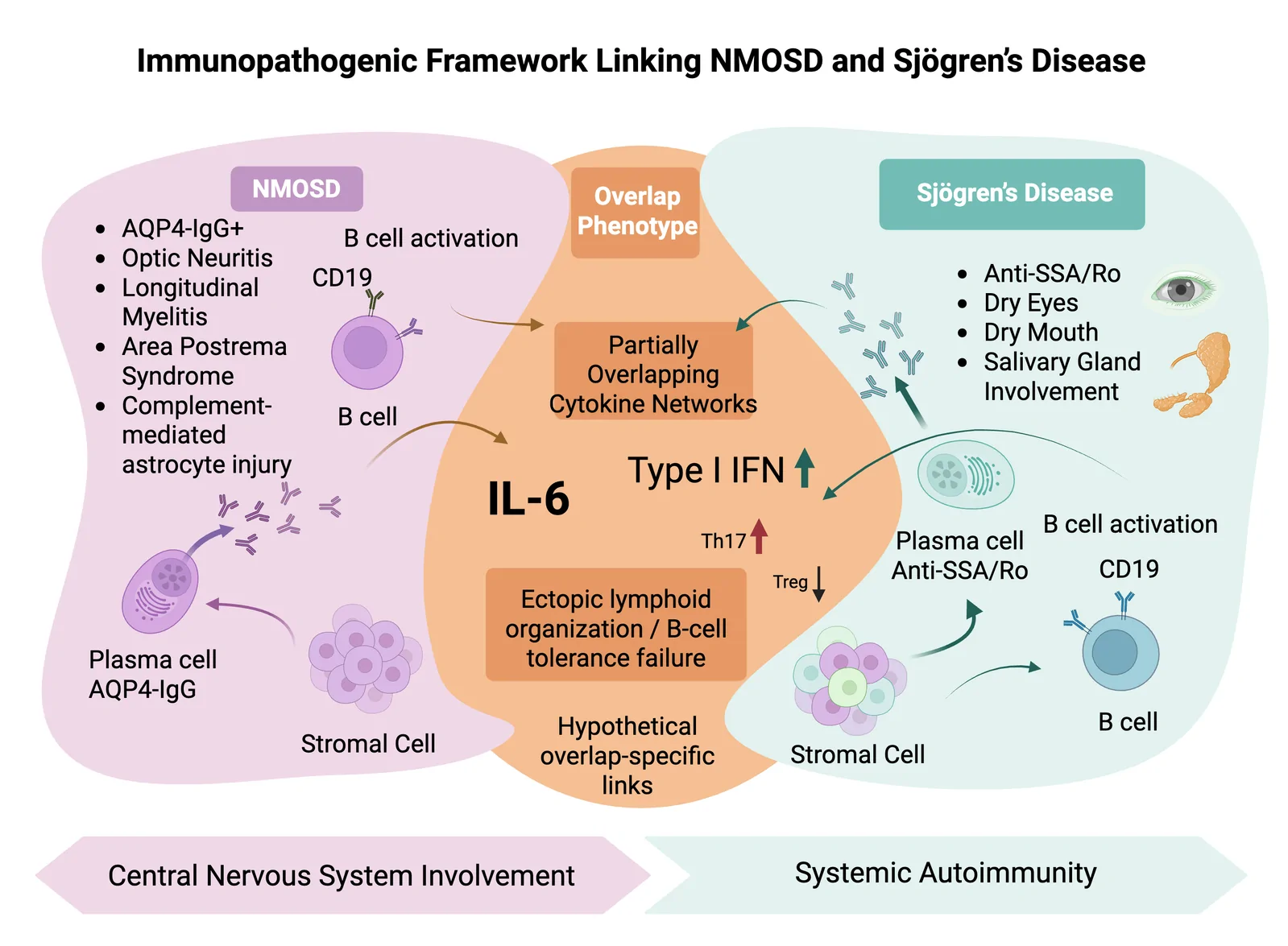
Integrated immunopathogenic model of partially overlapping mechanisms in neuromyelitis optica spectrum disorder (NMOSD) and Sjögren’s disease (SjD). Activation of BAFF-related B-cells, IL-6 signaling, and type I interferon may sustain systemic humoral autoimmunity in SjD and support survival of autoreactive B-cell lineages. In AQP4-IgG-positive NMOSD, circulating AQP4-IgG binds to astrocytic endfeet and triggers complement-mediated injury of astrocytes. T cells and innate immune cells can increase the production of cytokines, activation of the endothelium, and recruitment of inflammatory effector cells. Dysfunction of the BBB may allow circulating pathogenic antibodies and inflammatory mediators to access the CNS. This model should be viewed as a biologically plausible framework for NMOSD-SjD overlap rather than proof of a distinct disease entity. Created in BioRender. Hou, L. (2026). https://BioRender.com/v95ctyd.

Taken together, studies that mostly examined NMOSD and SjD as separate diseases suggest there may be several potentially intersecting pathways, including systemic B-cell-dominant autoimmunity, cytokine activation, complement-mediated injury of astrocytes, and dysfunction of the BBB (12, 34, 48–50, 53, 57–61). In this conceptual framework, SjD provides a systemic autoimmune context characterized by B cell activation, IFN/BAFF signaling, and ectopic lymphoid organization in some patients. In contrast, AQP4-IgG-positive NMOSD represents a CNS-specific antibody-mediated astrocytopathy (24) (**Figure 2**).

Importantly, this framework is inferential, and does not indicate that NMOSD-SjD overlap is a distinct disease entity, that SjD directly causes AQP4-IgG-mediated astrocyte injury, or that the proposed shared pathways can be used to define validated biomarkers for treatment selection. The main value of this framework is to organize current biological hypotheses and identify questions that require more direct investigations in well-characterized overlap cohorts.

## 5 Diagnostic challenges and differential diagnosis

### 5.1 When to suspect Sjögren’s disease in patients with NMOSD

Identifying underlying SjD in patients with NMOSD remains challenging, particularly when there are no overt symptoms of sicca (1, 63). Although xerophthalmia and xerostomia are classically associated with SjD, multiple cohort studies have demonstrated that neurological involvement may precede or even dominate the clinical presentation, thereby complicating screening and diagnosis (1, 2, 8, 15, 36).

Several clinical and serological characteristics may suggest the presence of SjD in patients with NMOSD: unexplained arthralgia or fatigue, cytopenias, antinuclear antibodies, anti-SSA/Ro antibodies, hypocomplementemia, renal tubular abnormalities, parotid enlargement, and other systemic autoimmune features (1, 10, 14, 63). Hypergammaglobulinemia may also be present, particularly in patients with classical systemic rheumatologic manifestations, but is not always present in patients with neurological presentations. Importantly, anti-SSA/Ro positivity may occur in patients who do not meet the ACR-EULAR criteria for SjD. In this setting, anti-SSA/Ro should be interpreted as evidence of serological autoimmunity that warrants further clinical and glandular assessment, not a diagnosis of SjD (31, 33).

Given these considerations, reliance on the presence of sicca alone may lead to the underdiagnosis of SjD in patients with NMOSD (7, 19, 64, 65). Therefore, baseline screening for concomitant connective tissue disease, including antinuclear antibodies (ANAs) and extractable nuclear antigen (ENA) antibodies (such as anti-SSA/Ro) is reasonable in patients with suspected or newly diagnosed NMOSD. However, comprehensive evaluation of SjD should be reserved for patients with positive serology, sicca symptoms, or other clinical or laboratory features of systemic autoimmunity (1, 2, 35).

### 5.2 Central nervous system involvement in SjD versus NMOSD

The differentiation of NMOSD from SjD with CNS involvement is challenging because patients with either condition may present with inflammatory demyelination (1, 2). Patients with SjD can present with diverse CNS manifestations, including optic neuritis, transverse myelitis, brainstem syndromes, and nonspecific lesions of white matter (15, 36, 37), that can suggest NMOSD, particularly when sicca is not present.

However, several important differences can aid in the differential diagnosis. Patients with NMOSD typically experience severe and relapsing attacks, longitudinally extensive transverse myelitis, bilateral or recurrent optic neuritis, area postrema syndrome, and AQP4-IgG seropositivity (4, 12). In contrast, CNS involvement in patients with SjD is usually more heterogeneous and may be less severe, with variable distribution of lesions and a lower frequency of relapse (25).

Patients with these conditions also have differences in CSF. Patients with NMOSD typically have mild pleocytosis with elevated levels of protein during acute attacks, and oligoclonal bands are less common than in MS (41, 66). Patients with SjD-related CNS disease have variable CSF findings that may be a consequence of systemic immune activation (12, 15, 25). Although no single characteristic is definitive, an integration of the attack phenotype, the status of AQP4-IgG and MOG-IgG, the distribution of MRI lesions, CSF findings, and SjD-related systemic features within a structured diagnostic framework can help to prevent misclassification and misdiagnosis (4, 35, 63, 64).

### 5.3 NMOSD versus MOG antibody–associated disease in patients with SjD

Although myelin oligodendrocyte glycoprotein antibody-associated disease (MOGAD) is very rare, it should also be considered in the differential diagnosis of patients with inflammatory demyelinating disorders, particularly in those with systemic autoimmunity (41, 42). Specifically, previous studies have reported MOG-IgG positivity in patients with SjD and other connective tissue diseases, and the clinical presentation of MOGAD may overlap with NMOSD and SjD with CNS involvement (66, 67).

It is critical to distinguish these diseases because they have differences in pathogenesis, and the patients have differences in prognosis and response to treatments. Thus, patients with MOGAD often have bilateral optic neuritis, alterations in the conus medullaris, and better recovery after acute attacks; patients with NMOSD tend to have a more severe and relapsing course of disease (41, 42, 66, 68, 69). Importantly, patients with MOGAD do not have AQP4-IgG positivity or astrocytopathy, underscoring the necessity of comprehensive antibody testing (41, 42).

When a patient presents with SjD and CNS demyelination, exclusive reliance on AQP4-IgG testing may be insufficient (41, 42). In such instances, testing for MOG-IgG should also be considered, particularly when the patient has atypical imaging results or a positive response to steroid therapy (41, 42). Failure to recognize MOGAD may lead to inaccurate prognostic counseling and inappropriate treatment decisions, because MOGAD and AQP4-IgG–positive NMOSD differ in disease course, relapse risk, and therapeutic response (66, 69).

### 5.4 Practical diagnostic approach to NMOSD–SjD overlap

**Figure 3:**
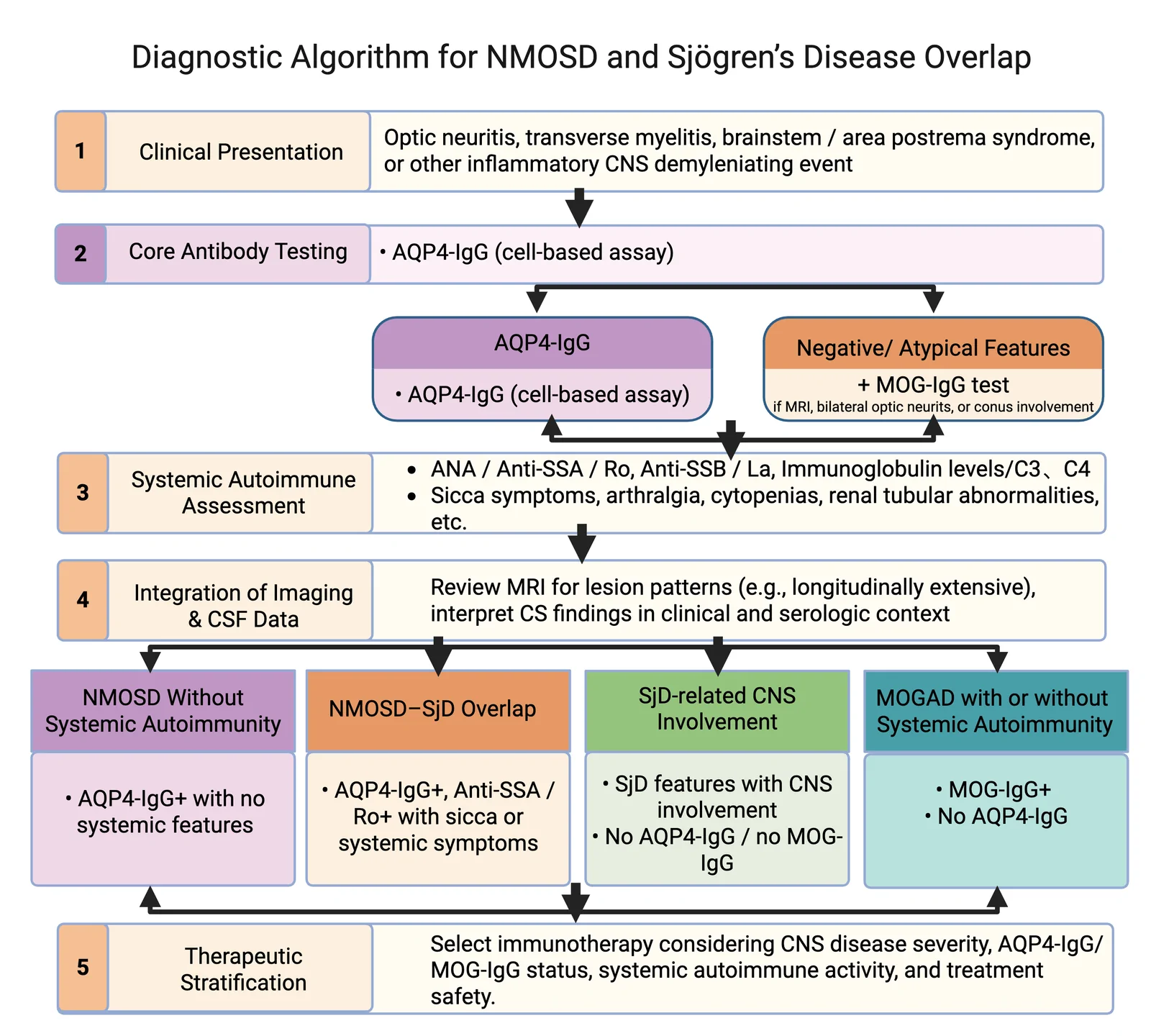
Diagnostic algorithm for patients presenting with inflammatory demyelinating disease and suspected overlap of neuromyelitis optica spectrum disorder (NMOSD) with Sjögren’s disease (SjD). The clinician should first classify the CNS inflammatory disorder by testing for the attack phenotype, AQP4-IgG, and MOG-IgG, distribution of lesions in MRI, and analysis of CSF. Evaluation for SjD should then proceed in parallel using serological screening, assessment of systemic autoimmunity, and objective glandular tests when indicated. Labial salivary gland biopsy should be reserved for patients with inconclusive serology or when histological confirmation would alter treatment, surveillance, or risk stratification. This stepwise strategy aims to avoid misclassification while considering diagnostic yield, disease severity, cost, and clinical impact. Created in BioRender. Hou, L. (2026). https://BioRender.com/k8uecsh.

The diagnostic challenges described above highlight the need for a structured and integrated approach for patients with suspected NMOSD-SjD overlap (4, 35, 64, 66, 70) (**Figure 3**). In clinical practice, the first priority is to establish whether the inflammatory demyelinating event fulfills the criteria for AQP4-IgG-positive NMOSD, MOGAD, or another CNS inflammatory disorder (4, 35, 66, 70). This requires determination of serum AQP4-IgG using a cell-based assay, MOG-IgG when clinically appropriate, MRI of the brain, spinal cord, and optic nerves according to presentation, and analysis of CSF during acute attacks (4, 35, 38, 66, 70). These tests should not be delayed by the evaluation of SjD, because early classification of a CNS disorder can directly influence the strategies to prevent relapse (27, 35).

Baseline assessments for concomitant systemic autoimmunity should include a focused clinical history and examination, and tests for ANAs and ENA antibodies (including anti-SSA/Ro and anti-SSB/La), complete blood count, immunoglobulins, C3/C4, renal function, and urinalysis (34, 49, 64). More extensive evaluation for SjD should be performed when patients have anti-SSA/Ro positivity, sicca, unexplained arthralgia or fatigue, cytopenias, hypergammaglobulinemia, hypocomplementemia, renal tubular abnormalities, parotid enlargement, pulmonary involvement, or other indications of systemic autoimmunity. Objective glandular assessment should be guided by the clinical and serological findings. The Schirmer test, ocular staining score, and unstimulated salivary flow are included in the 2016 ACR-EULAR classification criteria for SjD. Salivary gland ultrasound (SGUS) can identify structural abnormalities of the major salivary glands and may help determine whether further investigation is warranted. However, SGUS is not included in the 2016 ACR–EULAR criteria because its performance depends on operator expertise and scoring standardization, and normal results do not exclude SjD; SGUS should therefore not be regarded as a direct substitute for anti-SSA/Ro testing or labial salivary-gland biopsy (49, 64, 71).

Biopsy of the labial salivary gland should not be used as a first-line test for all NMOSD patients. The 2016 ACR-EULAR criteria consider focal lymphocytic sialadenitis with 1 or more foci per 4 mm^2^ as a highly weighted classification item that is particularly informative in patients with anti-SSA/Ro negativity or when classification remains uncertain. Biopsy should be considered when histopathological confirmation would affect diagnostic classification, long-term surveillance, or therapeutic management, and the specimen should be obtained and interpreted using standardized methods. The limitations of biopsy are that it is invasive, the sampling may be inadequate, and there is variability in the histopathological interpretation. For anti-SSA/Ro-positive patients with supportive objective glandular findings, biopsy may not be necessary to solely satisfy the classification criteria. Instead, persistent major salivary-gland enlargement or suspected lymphoma should prompt targeted imaging and sampling of clinically abnormal glands or lymph nodes (34, 49, 64). This tiered approach is summarized in **Table 2**.

**Table 2:** Essential and optional diagnostic tests for suspected NMOSD-SjD overlap (4, 27, 34, 35, 49, 61, 64, 82).

Diagnostic domain	Essential tests	Conditional or optional tests	Clinical rationale
CNS demyelinating disorder classification	Serum AQP4-IgG using a cell-based assay; serum MOG-IgG when the phenotype is atypical or AQP4-IgG is negative; MRI of brain, spinal cord, and optic nerves according to symptoms; CSF analysis during acute attacks	Repeat antibody testing if initial results are negative but clinical suspicion remains high; visual evoked potentials or optical coherence tomography when optic nerve involvement is uncertain	Establishes whether the patient has AQP4-IgG–positive NMOSD, MOGAD, SjD-related CNS disease, MS, or another inflammatory CNS disorder
Screening for systemic autoimmunity relevant to SjD	ANA, anti-SSA/Ro, anti-SSB/La, rheumatoid factor, serum IgG, C3/C4, ESR, CRP, complete blood count, liver and renal function, urinalysis, urine protein assessment	Cryoglobulins, serum free light chains, β2-microglobulin, complement monitoring, renal tubular acidosis evaluation when clinically indicated	Identifies systemic autoimmune features, humoral immune activation, cytopenias, renal involvement, hypocomplementemia, and lymphoproliferative risk. Anti-SSA/Ro positivity supports clinical suspicion, but does not independently establish SjD
Objective glandular assessment	Sicca symptoms, medication history, and dental and ocular history; Schirmer test, ocular staining score, or unstimulated whole salivary flow as clinically indicated	^a^SGUS using a standardized scoring system when available; ophthalmology or rheumatology assessment	Objective ocular and salivary tests for SjD classification. SGUS is a non-invasive adjunct but not a formal 2016 ACR-EULAR criterion, is operator-dependent, and does not independently confirm or exclude SjD
Labial salivary gland biopsy	Not required as a routine first-line test in all NMOSD patients	Consider when anti-SSA/Ro is negative or inconclusive and SjD remains clinically suspected, or when histopathological confirmation would alter classification, surveillance, or management	Focal lymphocytic sialadenitis ( ≥1 focus/4 mm^2^) is a highly weighted item in the 2016 ACR–EULAR criteria. Limitations include invasiveness, sampling adequacy, and variability in pathological interpretation; standardized acquisition and interpretation are required
Systemic organ assessment	History and examination for arthralgia, parotid enlargement, rash, Raynaud’s phenomenon, cytopenias, renal tubular dysfunction, pulmonary symptoms, and constitutional symptoms	Chest imaging, renal tubular function tests, salivary gland imaging, hematology evaluation, or tissue biopsy (depending on organ involvement)	Identifies extraglandular SjD manifestations that may influence long-term monitoring, infection, and treatment with immunosuppressants
Pre-treatment safety assessment	Complete blood count, liver and renal function, immunoglobulin levels, hepatitis B/C screening, tuberculosis screening, vaccination review	HIV testing, varicella-zoster status, meningococcal vaccination and prophylaxis when complement inhibition is considered, baseline assessment of infection	Supports safe selection of B-cell–targeted therapy, IL-6 receptor blockade, complement inhibition, or conventional immunosuppression

^a^SGUS should be regarded as an adjunct rather than a replacement for labial salivary-gland biopsy. When lymphoma is suspected because of persistent focal glandular enlargement, mass lesions, or lymphadenopathy, then targeted imaging and biopsy of the abnormal site are preferred.

## 6 Treatment of NMOSD-SjD overlap phenotype

### 6.1 Management of acute attacks

An acute attack of NMOSD is a neurological emergency that can lead to irreversible disability. Thus, a patient with NMOSD-SjD overlap should initially receive treatment for acute optic neuritis and transverse myelitis according to standard recommendations (27, 35, 72). The initial treatment with high-dose intravenous methylprednisolone remains a first-line therapy, and early escalation to plasma exchange or immunoadsorption can be given to patients with severe attacks or an inadequate response to steroid therapy (22, 72). Although management of these acute symptoms is generally similar for patients with the overlap phenotype, clinicians should recognize that systemic autoimmune activity in SjD (e.g., cytopenia, vasculitis, and systemic inflammation) may decrease tolerance to methylprednisolone, increase the risk of infection, and impair recovery (18, 19). This points to the need for early multidisciplinary assessment that balances the requirement for urgent treatment of the acute symptoms with systemic safety (18, 19).

However, the presence of systemic autoimmunity in a patient with NMOSD-SjD overlap should not delay NMOSD-specific treatment escalation when severe optic neuritis, longitudinally extensive transverse myelitis, or brainstem involvement is present (27, 72). AQP4-IgG positivity is a major pathogenic autoantibody that is related to complement-mediated injury of astrocytes and requires rapid suppression of inflammation (6, 12). In practice, a patient with the overlap phenotype should receive a broader assessment at baseline, which includes measurements of immunoglobulins, blood count, and hepatic function, rather than a fundamentally different algorithm (18, 19).

### 6.2 Biologics and conventional immunosuppression for prevention of long-term relapse

Prevention of long-term relapse is the central therapeutic goal in AQP4-IgG-positive NMOSD, because recurrent attacks and incomplete recovery can lead to neurological disability. For patients with NMOSD-SjD overlap, the primary therapeutic goal remains the prevention of NMOSD relapse. The presence of SjD should be considered during assessments of systemic disease, treatment contraindications, and safety monitoring, but it does not currently provide validated criteria for selecting a specific NMOSD maintenance therapy. No randomized controlled trials have specifically evaluated treatment for NMOSD-SjD overlap. Therefore, therapies that target prevention of relapse should be selected primarily according to evidence from AQP4-IgG-positive NMOSD, and SjD-related systemic manifestations should be managed according to established SjD principles (27, 28, 34, 49).

The introduction of targeted biologics has increased the number of therapeutics available for treatment of AQP4-IgG-positive NMOSD. The PREVENT trial showed that complement inhibition with eculizumab significantly reduced the risk of relapse, and ravulizumab (a long-acting C5 inhibitor) can also be used to prevent relapse in AQP4-IgG-positive NMOSD (73–75). Two recent trials support the use of IL-6 receptor blockade using satralizumab for treatment of NMOSD. The Phase 3 SAkuraSky trial found that satralizumab was effective as an add-on therapy and the Phase 3 SAkuraStar trial supported the use of satralizumab monotherapy (51, 54). In addition, the Phase 2/3 N-MOmentum trial found that targeting B-cells with inebilizumab (an anti-CD19 monoclonal antibody) decreased the risk of relapse in NMOSD. This drug also had favorable long-term safety and effectiveness in follow-up and real-world studies (76–78). Rituximab remains a widely used B-cell-depleting therapy, and may be a suitable option in settings where other biologics are unavailable, unaffordable, or unsuitable (27, 79). The evidence summarized above is from NMOSD trials and observational studies, rather than studies of treatments specifically designed for patients with NMOSD-SjD overlap.

A practical approach is to select a relapse-prevention therapy primarily according to AQP4-IgG status, history of relapse, severity of attack(s), residual disability, comorbidities, treatment safety, drug access, route of administration, and patient preference. Clinical trials of NMOSD have supported the use of complement inhibitors (satralizumab and inebilizumab), whereas rituximab, azathioprine, and mycophenolate mofetil are used off-label or as pragmatic alternatives according to local availability and clinical circumstances (27, 28, 51, 54, 73–79). Systemic SjD activity may influence the overall immunosuppressive regimen and safety monitoring; however, IL-6 activity, broad production of autoantibodies, and B-cell hyperactivity are not validated biomarkers for selecting a specific biologic in patients with NMOSD-SjD overlap. Similarly, evidence from the treatment of SjD should not be interpreted as proof of efficacy for patients with the overlap disease. The level of evidence for NMOSD, regulatory or off-label status of different therapeutics, availability of overlap-specific evidence, and safety considerations for these therapies are summarized in **Table 3**.

**Table 3:** Therapeutic approaches, evidence and regulatory status, and safety considerations for NMOSD-SjD overlap (27, 28, 34, 49, 51, 54, 73–79).

Therapeutic approach	Main immunological target	^a^NMOSD evidence/regulatory status and overlap-specific evidence	Key safety and practical considerations
High-dose intravenous methylprednisolone	Broad anti-inflammatory effect	Standard first-line treatment for acute NMOSD attacks. No evidence from comparative trials that evaluated acute treatment for NMOSD–SjD overlap	Monitor blood pressure, glucose, infection, psychiatric symptoms, gastrointestinal complications, and systemic SjD activity
Plasma exchange or immunoadsorption	Removal of pathogenic antibodies and inflammatory mediators	Established escalation treatment for severe or corticosteroid-refractory NMOSD attacks. No evidence from overlap-specific comparative trials	Requires vascular access; monitoring of hemodynamic stability, coagulation status, infection, and local approval/availability
Eculizumab	Terminal inhibition of C5	Supported by randomized NMOSD trial evidence and approved for adult AQP4-IgG-positive NMOSD in multiple jurisdictions. No evidence from overlap-specific trials	Meningococcal vaccination and monitoring of infection; cost, access, infusion burden, and local approval/availability
Ravulizumab	Long-acting terminal inhibition of C5	Supported by NMOSD clinical-trial evidence and approved for adult AQP4-IgG-positive NMOSD in some jurisdictions. No evidence from overlap-specific trials	Similar to Eculizumab
Satralizumab	IL-6 receptor blockade	Supported by randomized NMOSD trials and approved for AQP4-IgG-positive NMOSD in multiple jurisdictions. No evidence from overlap-specific trials	Monitor neutropenia, liver enzymes, lipid profile, infection, gastrointestinal complications, and ‘masking’ of CRP
Tocilizumab	IL-6 receptor blockade	Off-label for NMOSD, supported mainly by observational and comparative studies. No evidence from overlap-specific trials	Monitor neutropenia, liver enzymes, lipid profile, infection, gastrointestinal complications, and ‘masking’ of CRP
Inebilizumab	CD19-positive B-cell lineage, including plasmablasts	Supported by randomized NMOSD trials and approved for adult AQP4-IgG-positive NMOSD in multiple jurisdictions. No evidence from overlap-specific trials	Monitor immunoglobulins, infections, infusion reactions, hepatitis B status, and prolonged B-cell depletion
Rituximab	CD20-positive B cells	Off-label for NMOSD in many jurisdictions, supported by observational and comparative clinical evidence. Limited case-based evidence in SjD patients with CNS demyelination. No evidence from overlap-specific randomized trials	Monitor B-cell repopulation, immunoglobulins, late-onset neutropenia, hepatitis B reactivation, infusion reactions, and infection
Azathioprine or mycophenolate mofetil	Broad lymphocyte suppression	Off-label conventional maintenance therapies supported mainly by observational NMOSD evidence. Possibly suitable as pragmatic alternatives when approved biologics are unavailable or unsuitable. No evidence from overlap-specific comparative trials	Slower onset; need to monitor leukopenia, liver toxicity, infection, gastrointestinal intolerance, reproductive issues, and drug interactions
Treatment de-escalation or discontinuation	Reduction of cumulative immunosuppression	Evidence regarding relapse after discontinuation is mainly from AQP4-IgG-positive NMOSD, not overlap-specific cohorts. No evidence of validated overlap-specific discontinuation criteria	Requires individualized risk-benefit assessment, sustained stability, close monitoring, and patient counseling

^a^Regulatory approval varies among countries and regions. “Overlap-specific evidence” refers to studies that separately evaluated patients with NMOSD and SjD. No randomized trial has specifically evaluated maintenance therapy in this population.

### 6.3 Safety monitoring and systemic considerations beyond prevention of relapse

Management of NMOSD-SjD overlap should extend beyond the prevention of neurological relapse, and should include assessment of systemic disease, prevention of infection, and management of long-term safety. Baseline evaluations should include measurements of complete blood count, markers of liver and renal function, serum immunoglobulins, screening for hepatitis B/C and tuberculosis, review of vaccination history, and assessment of prior and current use of immunosuppressants (27, 28, 34, 49). These considerations are particularly important in overlap patients because SjD can be accompanied by cytopenias, hypocomplementemia, renal tubular involvement, pulmonary disease, cryoglobulinemia, and lymphoproliferation.

Safety during treatment should consider the specific mechanism of the therapeutic. Thus, B-cell-depleting or B-cell-targeted therapies require monitoring for hypogammaglobulinemia, late-onset neutropenia, reactivation of hepatitis B, and recurrent infections. IL-6 receptor blockade requires determinations of neutrophils, liver enzymes, lipid abnormalities, gastrointestinal complications, and masking of inflammatory markers (especially CRP and ESR). Therapies that inhibit complement should be accompanied by meningococcal vaccination and patient education regarding meningococcal infection, and antimicrobial prophylaxis according to local practice. Conventional immunosuppressants require monitoring for leukopenia, liver toxicity, infection, gonadal function, and drug-specific adverse effects. Treatment selection should also consider drug availability, reimbursement, route and frequency of administration, adherence, and patient preference (27, 28).

### 6.4 Treatment pitfalls: misclassification, inappropriate therapies, and risk of discontinuation

Misclassification remains a major therapeutic pitfall in the treatment of NMOSD-SjD overlap. Treating AQP4-IgG-positive NMOSD as MS (whose symptoms can be similar) can expose patients to ineffective or potentially harmful disease-modifying therapies (4, 22). However, for patients with the overlap disease, clinicians may fail to recognize coexisting SjD, and this may lead to incomplete assessment of systemic risk, insufficient monitoring of infection and cytopenia, under-recognition of extraglandular disease, and selection of inadequate therapeutics. Similarly, a lack of autoantibody testing may lead clinicians to attribute demyelinating events to CNS involvement of SjD and delay the initiation of therapy that can prevent NMOSD relapse.

A second therapeutic pitfall to be avoided is the failure to consider MOGAD in SjD patients who present with demyelination (41, 42). Although there are similarities in the immunosuppressive treatments given SjD patients with MOGAD and patients with AQP4-IgG-positive NMOSD, these two groups differ in prognosis and risk of relapse. Thus, the use of long-term biologics for patients with AQP4-IgG-positive NMOSD may be unsuitable (66, 69). Therefore, comprehensive antibody testing is essential before implementation of long-term therapy (41, 42).

Finally, discontinuation or de-escalation of immunosuppressive therapeutics should be approached cautiously in patients with AQP4-IgG-positive NMOSD. Available evidence regarding relapse after treatment withdrawal derives primarily from broad cohorts of patients with NMOSD rather than patients with NMOSD-SjD overlap. A case report described a single patient with both conditions who had severe relapse after cessation of immunosuppressive therapy, although this does not establish that coexisting SjD independently increases the risk of relapse after treatment withdrawal (27, 80, 81). Decisions regarding discontinuation of treatment should therefore follow NMOSD-specific guidelines and be based on individualized assessments of serostatus, previous severity of attacks, duration of remission, treatment toxicities, and patient preference. At present, there is no validated overlap-specific de-escalation strategy.

### 6.5 Framework for pragmatic management of patients

In summary, the management of NMOSD-SjD overlap should employ standard measures to prevent NMOSD relapse and incorporate SjD-informed systemic assessment and monitoring of safety. For patients with AQP4-IgG-positive NMOSD, approved biologics or those with significant evidence of efficacy and safety that target complement, IL-6 signaling, or B-cell lineages should be considered when available and appropriate. The presence of SjD should not delay or replace NMOSD-specific prevention of relapse or dictate selection of a specific NMSOD biologic; instead, it should primarily inform the management of systemic manifestations, potential contraindications, and safety. Patients with the overlap disease also require monitoring and treatment of infections, measurements of cytopenia and hypogammaglobulinemia, and long-term monitoring for involvement of systemic organs or lymphoproliferative complications. In settings where approved biologics are unavailable or unaffordable, rituximab, azathioprine, or mycophenolate mofetil are pragmatic alternatives, but the limitations of these traditional therapeutics must be recognized.

## 7 Future directions and conclusions

The recognition of NMOSD-SjD overlap has increased substantially over the past decade, yet important knowledge gaps remain. Future research should move beyond simple descriptions of the coexistence of these two autoimmune diseases toward more precise characterization of the clinical phenotypes, temporal relationships, immunological profiles, and treatment outcomes. It is unknown whether NMOSD-SjD overlap is a distinct disease entity. Thus, based on current evidence, it is more appropriate to regard the overlap disease as a clinically meaningful phenotype that is characterized by AQP4-IgG-mediated astrocytopathy and systemic autoimmunity.

One priority for future studies of the overlap disease is the identification of biomarkers that can stratify patients according to the risk of neurological relapse, systemic autoimmune activity, and safety of different therapeutics. Beyond conventional autoantibodies, these biomarkers may include indicators of B-cell and plasmablast phenotypes, BAFF and IL-6 pathway signaling, type I IFN activity, complement activation, and barrier dysfunction. However, initial studies should interpret these biomarkers as research priorities rather than validated clinical tools. Longitudinal studies that integrate immunophenotyping using these biomarkers, neuroimaging results, measurement of systemic SjD activity, and treatment outcomes are needed to confirm the use of these biomarkers for prediction of relapse, systemic complications, and treatment-related adverse events (12, 27, 34, 49, 53, 57).

Prospective cohort studies and registry-based analyses are additional priorities. Most current evidence regarding NMOSD-SjD overlap derives from studies of retrospective cohorts, administrative databases, case series, or secondary interpretation of NMOSD trials, and many of these studies did not systematically classify SjD or analyze overlap patients as a predefined subgroup. Future studies should use standardized definitions of AQP4-IgG-positive NMOSD and MOGAD and the ACR-EULAR classification of SjD. It is also necessary to determine systemic disease activity, consider the efficacy of previous treatments, assess relapse and progression of disability, and safety endpoints. Such data are essential for determining the effect of overlap status on prognosis, treatment response, and risks from de-escalation of autoimmune treatments.

Future clinical trials and real-world studies of NMOSD-SjD overlap should consider SjD status, the presence of anti-SSA/Ro antibodies, features of systemic autoimmunity, and prior use of immunosuppressants. Given the difficulty of studying patients with rigorously defined NMOSD-SjD overlap, subgroup analyses using NMOSD registries are more feasible than conducting randomized trials. Analyses of these registries should clarify whether coexisting SjD influences the risk of relapse, the selection and efficacy of different biologics, the risk of infection, cytopenias, and hypogammaglobulinemia.

In conclusion, the co-occurrence of NMOSD and SjD is clinically important and unlikely due to chance, although there is no proof of a causal relationship or evidence of overlap-specific biomarkers. Current evidence supports recognition of NMOSD-SjD overlap as a clinically meaningful phenotype in which there is co-occurring systemic B-cell-dominant autoimmunity, activation of cytokines, complement-mediated injury of astrocytes, and BBB dysfunction. Recognition of this phenotype has practical implications for the diagnosis and classification of these patients, assessment of systemic risk, selection of treatments, and monitoring of long-term safety. An integrated multidisciplinary approach that accounts for AQP4-IgG-mediated CNS disease and SjD-related systemic autoimmunity should attempt to improve classification and individualized management while avoiding overinterpretation of the currently limited evidence.
